# Electrospun Polyacrylonitrile/Polyvinylidene Difluoride Bilayer Promoting the Uniform Lithium Deposition/Stripping in the Zero-Excess Lithium Metal Batteries

**DOI:** 10.1021/acsami.5c25604

**Published:** 2026-03-27

**Authors:** Yu-Jun Wei, Ai-Ling Huang, Hao-Yu Ku, Hsiang-Sheng Wei, Chi-Yu Lai, Chi-Chang Hu

**Affiliations:** † Department of Chemical Engineering, 34881 National Tsing Hua University, Hsin-Chu 300044, Taiwan; ‡ College of Semiconductor Research, National Tsing Hua University, Hsin-Chu 300044, Taiwan; § College of Sustainability, National Tsing Hua University, Hsin-Chu 300044, Taiwan

**Keywords:** zero-excess lithium metal batteries, electrospinning, lithium deposit morphology, inorganic-rich solid electrolyte interphase, polyacrylonitrile, poly(vinylidene difluoride)

## Abstract

The zero-excess lithium metal batteries (ZELMBs) offer a higher energy density and better manufacturing safety compared with the conventional LMBs. However, the practical application of such cells is hindered by the severe dendrite growth originating from the uneven Li^+^ distribution at the copper substrate. Here, we combine a top layer of polyacrylonitrile (PAN) and a bottom layer of poly­(vinylidene difluoride) (PVDF), fabricated by electrospinning, to serve as an artificial solid electrolyte interphase (ASEI) promoting membrane, denoted as Cu@PAN + PVDF, for effectively dealing with this challenge. This configuration facilitates the desolvation and uniform flux of Li^+^, leading to form an inorganic-rich SEI layer to favor the uniform lithium deposition. In contrast, reversing the layer order (i.e., PVDF as the top layer and PAN as the bottom layer, denoted as Cu@PVDF + PAN) results in a high nucleation barrier and an uneven lithium deposit. The morphological evolution is further examined using a newly designed half-stripping experiment where lithium is plated and partially stripped at 1 mA cm^–2^ for 1 and 0.5 h, respectively. The Cu@PAN + PVDF electrode maintains a dense, uniform surface, whereas Cu@PVDF + PAN exhibits midlayer voids and disconnected “dead Li”, indicating the uneven delithiation. The Cu@PAN + PVDF||Li half-cell achieves over 160 cycles with a Coulombic efficiency (CE) exceeding 95%, outperforming the Cu@PVDF + PAN||Li (100 cycles) and bare Cu||Li (110 cycles) cells. This work identifies layer orientation as a governing parameter for the ASEI design and introduces a practical half-stripping methodology for evaluating the interfacial reversibility of the negative electrode in ZELMBs.

## Graphical abstract

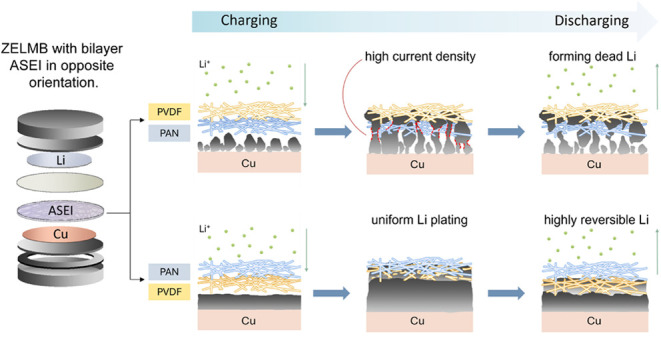

## 1 Introduction

Rechargeable batteries with a high energy density have become one of the most critical technologies of the 21st century.^1^ Among various energy storage systems, lithium-ion batteries (LIBs) have attracted significant attention due to their excellent cycling stability and energy density.^2,3^ However, the theoretical capacity of a common negative electrode material, graphite, of LIBs is limited to 372 mAh g^–1^, and there is an urgent need in developing next-generation energy storage systems with higher energy densities than LIBs.^4^ Hence, lithium metal batteries (LMBs) have been reproposed to be a promising candidate to replace LIBs, owing to the high theoretical specific capacity (3,860 mAh g^–1^), low density, and ultralow electrochemical potential (−3.04 V vs the standard hydrogen electrode) of metallic lithium used as the negative electrode.^1,5^ However, the heavy reliance on lithium with a high activity poses the high cost and safety risks, limiting the commercial viability of LMBs.^6^ To address these challenges, the concept of zero-excess lithium metal batteries (ZELMBs), also denoted as “anode-free” or “lithium–metal-free” lithium metal batteries, is proposed.

Compared to LMBs, ZELMBs exhibit a higher energy density, a more reduced manufacturing cost, and higher production safety due to the absence of metallic lithium.^7^ Unfortunately, due to the significant lattice mismatch between metallic lithium and copper, the heterogeneous nucleation of Li on Cu commonly results in a high nucleation energy barrier, leading to uneven Li^+^ flux and nonuniform plating morphology.^8,9^ Such inhomogeneous deposition probably accelerates the dendritic growth, induces repeated SEI cracking, and increases the continuous parasitic reactions. Moreover, freshly exposed Li and newly generated surface area further exacerbate the electrolyte decomposition, consuming both active Li and the electrolyte. Ultimately, the cycle life of ZELMBs is shortened.^10,11^ Hence, how to improve the interfacial compatibility and Li^+^ distribution on the Cu foil surface becomes crucial to the practical application of ZELMBs.^12^

To address the issue, extensive efforts have been devoted to enhance the electrolyte–electrode interphase, including the fabrication of artificial solid electrolyte interphases (ASEIs). This strategy aims to increase the stability of the SEI layer via (1) integrating an artificial layer with a native SEI or (2) introducing a coating layer between separators and the native SEI (i.e., in situ and ex situ methods).^13^ The former method usually uses the film-forming additives to regulate the solvation structure, generating an inorganic-rich SEI.^14–16^ The resulting inorganic components, such as Li_3_N, LiF, and Li_3_PO_4_, possess high ionic conductivity and interfacial energy, reducing the lithium nucleation barriers and dendrite growth.^17^ Despite these advantages, the in situ-formed ASEIs are limited by the electrolyte depletion, inhomogeneity, and lack of thickness control.^18^ Even worse, the electrolyte-derived SEIs are usually mechanically fragile and prone to fracture under the volume fluctuations during the battery cycling.^19,20^ To overcome this challenge, organic components, especially polymers, have been introduced as interlayers via the ex situ fabrication.^21^ The ex situ coating methods provided a precise control over thickness and uniformity,^22^ and the polymers take the advantage of excellent mechanical properties. Moreover, their functional groups, such as amide, nitrile, carbon–fluorine, and hydroxy groups, are able to act as lithiophilic sites, promoting the Li^+^ flux uniformity.^23–25^ For example, Zhang et al.^26^ reported a PVDF-based inorganic/polymer hybrid artificial SEI formed via a chemical lithiation-induced defluorination process, which enables in situ generation of highly dispersed LiF nanofillers with a tunable content throughout the polymer matrix. This uniform LiF/PVDF hybrid layer provides abundant Li^+^ transport pathways and improves the interfacial kinetics, thereby enhancing the cycling stability and Coulombic efficiency. Unfortunately, many conventional ex situ methods, including spin coating, vapor deposition, or electroplating, often suffer from the issues of low scalability, large material waste, and high cost.^13,21^

As a result, the electrospinning technique with process simplicity, scalability, and relatively low cost has emerged as a promising method for fabricating/modifying the ASEI layers.^27^ The resulting membranes exhibit high porosity, which promotes the electrolyte uptake and enhances the ionic conductivity.^28^ In addition, their three-dimensional fibrous structure also accommodates the volume changes during cycling, reducing the mechanical failure.^29^ For instance, Ku et al.^30^ reported an electrospun PAN/TiO_2_ membrane coated on Cu current collectors. The high-surface-area PAN nanofibers with abundant nitrile groups could provide lithiophilic sites and enhance the electrolyte affinity, while the TiO_2_ decoration lowered the interfacial reaction barrier, enabling uniform Li deposition on Cu.

Building on the above studies, electrospun polymeric fibrous interlayers represent a practical and effective strategy to engineer the Cu current collector interface for ZELMBs. While numerous ASEI designs have been demonstrated using a single polymer system, the rational construction of multilayer fibrous ASEIs that integrate complementary functions from different polymers has been far less explored. Motivated by this gap, we selected polyacrylonitrile (PAN) and poly­(vinylidene difluoride) (PVDF) as our materials to fabricate an electrospun bilayer ASEI-promoting membrane. Here, PAN is expected to offer the high ionic conductivity and superior chemical stability,^31–33^ while PVDF has attracted considerable attention in the field of LIBs/LMBs due to its high-dielectric constant and excellent lithiophilicity arising from its abundant C–F bonds.^34,35^ Both materials have been reported to be effective in forming the inorganic-rich SEI to facilitate the uniform lithium deposition when they were utilized as the negative electrode-coating layer.^36,37^ Note that the strong electric field and stretching forces involved in electrospinning have been found to induce the formation of the β-phase of PVDF, which exhibits the highest dipole moment among its crystalline phases.^38^ The enhanced polarity and piezoelectric properties of β-PVDF make it particularly attractive for the energy storage applications.^39^ This again highlights the importance of an electrospinning process that enabled the crystalline control of materials through simple and low-cost processes.

This work demonstrated a bilayer electrospun membrane composed of PAN and β-PVDF as an ASEI-promoting layer on the negative electrode side of the ZELMBs. The nitrile groups in PAN were expected to facilitate the Li^+^ transport and desolvation process, while the C–F bonds in PVDF could regulate the Li^+^ flux and reduce the nucleation energy barrier on the copper current collector. These synergistic effects are believed to enable a highly uniform morphology of lithium deposits. Notably, the configuration with PAN as the top layer and PVDF as the bottom layer (denoted as Cu@PAN + PVDF) significantly enhanced the cycling stability. The Cu@PAN + PVDF||Li half-cell exhibited superior long-term cycling stability with a Coulombic efficiency above 95% over 160 cycles at 1 mA cm^–2^, in contrast to the Cu||Li cell under the same testing conditions (its CE was below 95% after 110 cycles). Furthermore, with prelithiating the Cu@PAN + PVDF negative electrode for an N/P ratio = 1.3, the Li/Cu@PAN + PVDF||LFP full cell achieves 57.68% capacity retention after 200 cycles, whereas Li/Cu||LFP retains only 27.7%. These results indicate that the dual-polymer ASEI-promoting membrane effectively stabilizes the interphase and enables the formation of an ultrathin, uniform Li deposit, which is essential for the ZELMB application.

## 2 Experimental Section

### 2.1 Preparation of Polymer Nanofibers

Polyacrylonitrile (PAN, *M*_w_ = 150,000 g mol^–1^, Sigma-Aldrich) was dissolved in *N*,*N*-dimethylformamide (DMF, Sigma-Aldrich) to form a 10 wt % solution. Separately, 10 wt % poly­(vinylidene difluoride) (PVDF, Kynar HSV1800) was dissolved in a mixed solvent of *N*-methyl-2-pyrrolidone (NMP) and acetone at 3:1 (v/v). No additional adjustment was made in this study since the volume ratio of solvent has already been optimized by the Abrha groups.^38^ Both solutions were stirred at 700 rpm for 6 h to ensure complete dissolution.

Electrospinning was conducted at an applied voltage of 12.5 kV using a 5 mL syringe equipped with a 0.24 mm inner diameter needle. The distance between the needle tip and the collector was set to 16 cm for PAN and 13 cm for PVDF. Then, the fibers were collected on soft baking paper mounted on a rotating drum collector. The resulting free-standing membranes were then prewetted with a small amount of electrolyte and subsequently laminated onto the Cu current collector prior to cell assembly. Electrolyte prewetting facilitates the conformal contact and improves the interfacial adhesion between the porous fibrous network and the Cu surface. The electrode surface with various fibrous membranes was denoted as Cu@PAN, Cu@PVDF, Cu@PVDF + PAN, and Cu@PAN + PVDF. Notably, PAN + PVDF and PVDF + PAN were obtained from the same dual-layer membrane (PAN/PVDF), with the two configurations distinguished by the orientation during cell assembly. The Cu@PAN + PVDF configuration refers to membranes with PAN facing the other electrode (top) and PVDF in contact with the current collector (bottom), whereas Cu@PVDF + PAN represents the reverse orientation.

Detailed parameters of the electrospinning process for each sample are given in Table S1. The thickness of the electrospun membranes was measured using a digimatic micrometer (Mitutoyo, Japan). To prevent the deformation of porous fibrous mats during the measurement, each membrane was sandwiched between two backing papers. The membrane thickness was obtained by subtracting the thickness of the backing papers from the measured total thickness. All corrected values are reported in the Supporting Information (Table S1 and Figure S1) (Figure 1).

**Figure 1:**
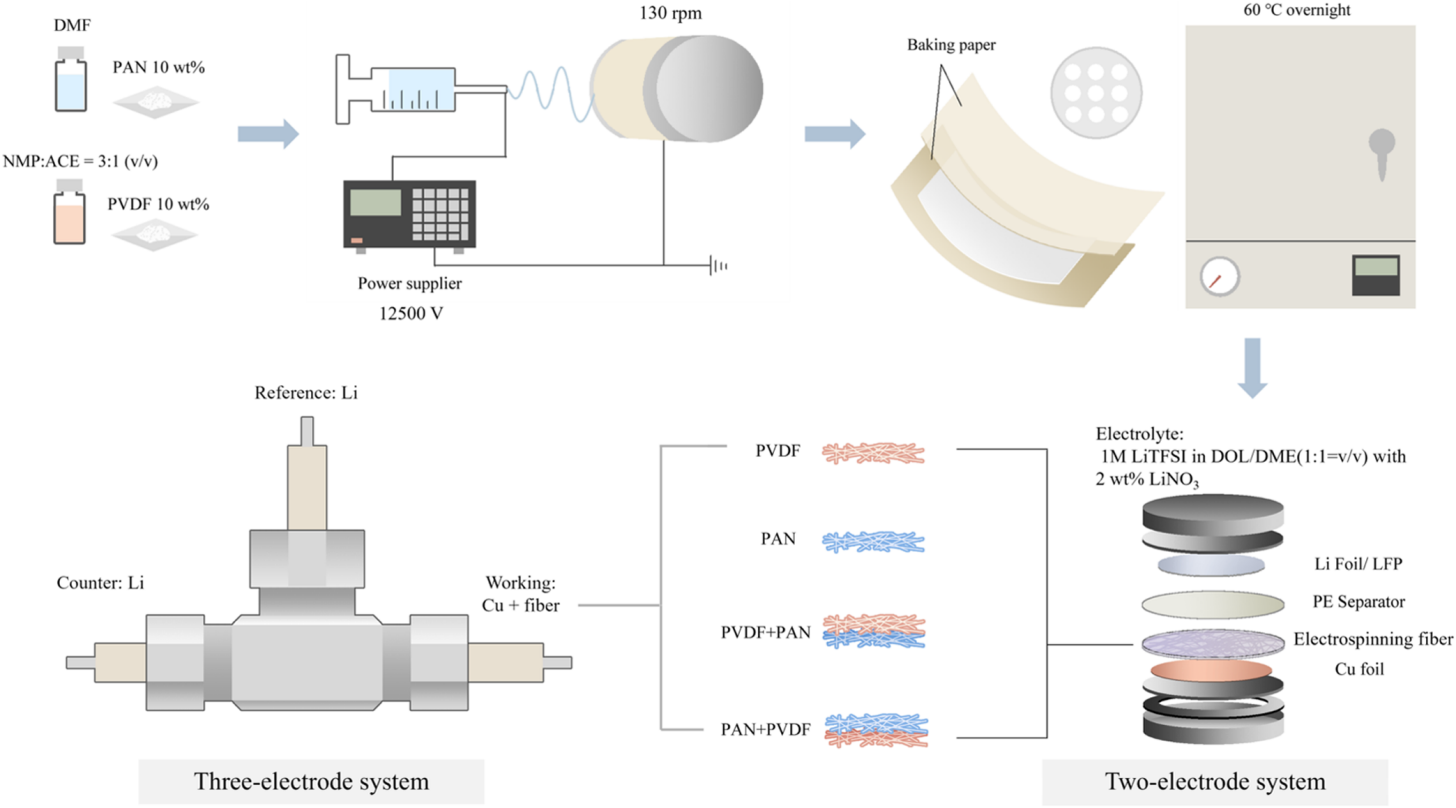
Schematic illustration of processing the polymer fibers and assembling the zero-excess button cell batteries.

### 2.2 Characterization of Electrospun Fibers

The morphology of electrospun PAN, PVDF, and PAN/PVDF fibrous membranes, as well as the Li deposits, was examined by scanning electron microscopy (SEM; SU-8010, Hitachi, Japan). The chemical structure of PVDF was analyzed using Fourier transform infrared spectroscopy (FTIR; Thermo Fisher Scientific, USA).

The porosity of the electrospun membranes was determined by a gravimetric method. Membranes were dried and weighed to obtain the dry mass (*W*_dry_). The samples were then fully immersed in a 99.5% ethanol until saturation. Afterward, they were gently blotted with filter paper to eliminate the excess surface liquid before weighing to obtain the wet mass (*W*_wet_). The porosity (ε) was obtained on the basis of 1:^40^

1 $$\varepsilon \left(\%\right)=\frac{\frac{W_{\mathrm{wet}}-W_{\mathrm{dry}}}{\rho_{\mathrm{solvent}}}}{\frac{W_{\mathrm{wet}}-W_{\mathrm{dry}}}{\rho_{\mathrm{solvent}}}+\frac{W_{\mathrm{dry}}}{\rho_{\mathrm{polymer}}}}\times 100\%$$

where ρ_solvent_ is the density of the wetting solvent, and ρ_polymer_ is the bulk density of the polymer.

The electrolyte uptake (EU) of the membranes was estimated using 2:

2 $$\mathrm{EU}\left(\%\right)=\frac{W_{t}-W_{0}}{W_{0}}\times 100$$

where *W*_0_ and *W*_*t*_ denote the membrane mass before and after electrolyte absorption, respectively. After being soaked, the membranes were gently blotted with filter paper prior to weighing to remove excess electrolyte on the surface.

### 2.3 Cell Assembling

CR2032 coin cells were assembled in an argon-filled glovebox (H_2_O < 10 ppm, O_2_ < 10 ppm) for electrochemical testing in the two-electrode configuration. Cu||Li half cells were constructed using the lithium foil (12 mm diameter) as the counter and reference electrodes, and the copper foil (14 mm diameter) with/without the electrospun ASEI-promoting membrane (16 mm) was the working electrode. A 25 μm-thick polyethylene (PE) separator (Asahi Chemical, Japan) was placed between the electrodes to prevent short-circuiting.

Cu||LFP full cells were assembled using the same procedure, except that the positive electrode was replaced with the LiFePO_4_-coated electrode (LFP, Ubiq Technology Co., Taiwan) with a mass loading of 11.52 mg cm^–2^ (areal capacity of 1.6 mAh cm^–2^). Each cell was filled with an electrolyte (50 μL) composed of 1 M LiTFSI in DOL/DME (1:1 v/v) with 2 wt % LiNO_3_. The water content in the electrolyte was confirmed to be below 5 ppm by using a Karl Fischer Coulometer (Metrohm, Switzerland). In this work, we intentionally employed an excess electrolyte volume to isolate and highlight the effect of ASEI-promoting membranes on the interfacial chemistry and Li deposition behavior.

For the three-electrode measurements, a custom-designed Swagelok-type cell was used, with lithium metal serving as the reference electrode and all other materials being identical to the coin cell configuration.

### 2.4 Electrochemical Measurements

The lithium-ion transference numbers (*t*_Li+_) were estimated by 3:^41^

3 $$t_{Li^{+}}=\frac{I_{s}\left(\Delta V-I_{0}R_{0}\right)}{I_{0}\left(\Delta V-I_{s}R_{s}\right)}$$

where Δ*V* is the applied voltage of 10 mV. *I*_0_ and *I*_S_ are the initial and steady-state currents measured via the chronoamperometric method. *R*_0_ and *R*_S_ refer to the initial and steady-state interfacial resistance of Li||Li cells, respectively, measured by electrochemical impedance spectroscopic (EIS). Both cycling voltammetric (CV) and galvanostatic charge–discharge (GCD) tests were conducted under the three-electrode system. CV was measured in the cell voltage window between 3.0 and 0.02 V for the first cycle and between 3.0 and −0.05 V in the second cycle at a scan rate of 1 mV s^–1^. GCD was performed at a current density of 1 mA cm^–2^ with an area capacity of 1 mAh cm^–2^ through a WBCS3000L battery testing system (WonATech, Korea).

## 3 Results and Discussion

### 3.1 Morphology and Chemical Structure of Fibers

As a prerequisite for evaluating the functional performance, the electrospun fibrous membrane was examined by SEM to verify its structural uniformity. As shown in Figure 2a,b, both PAN and PVDF membranes exhibit bead-free morphologies, indicating a uniform and defect-minimized fibrous network. Notably, the PAN fibers show an average diameter (∼700 nm) larger than that of PVDF (∼340 nm). Consistent with this morphology, the PAN membrane presents a larger pore feature and higher porosity (78%) than PVDF (59%). As summarized in Figure S1, the higher porosity correlates with a higher electrolyte uptake for the PAN fibrous membrane. This is expected to facilitate the electrolyte infiltration and provide the continuous ion-transport pathways within the porous network, thereby benefiting ionic transport through the interlayer.^42^

**Figure 2:**
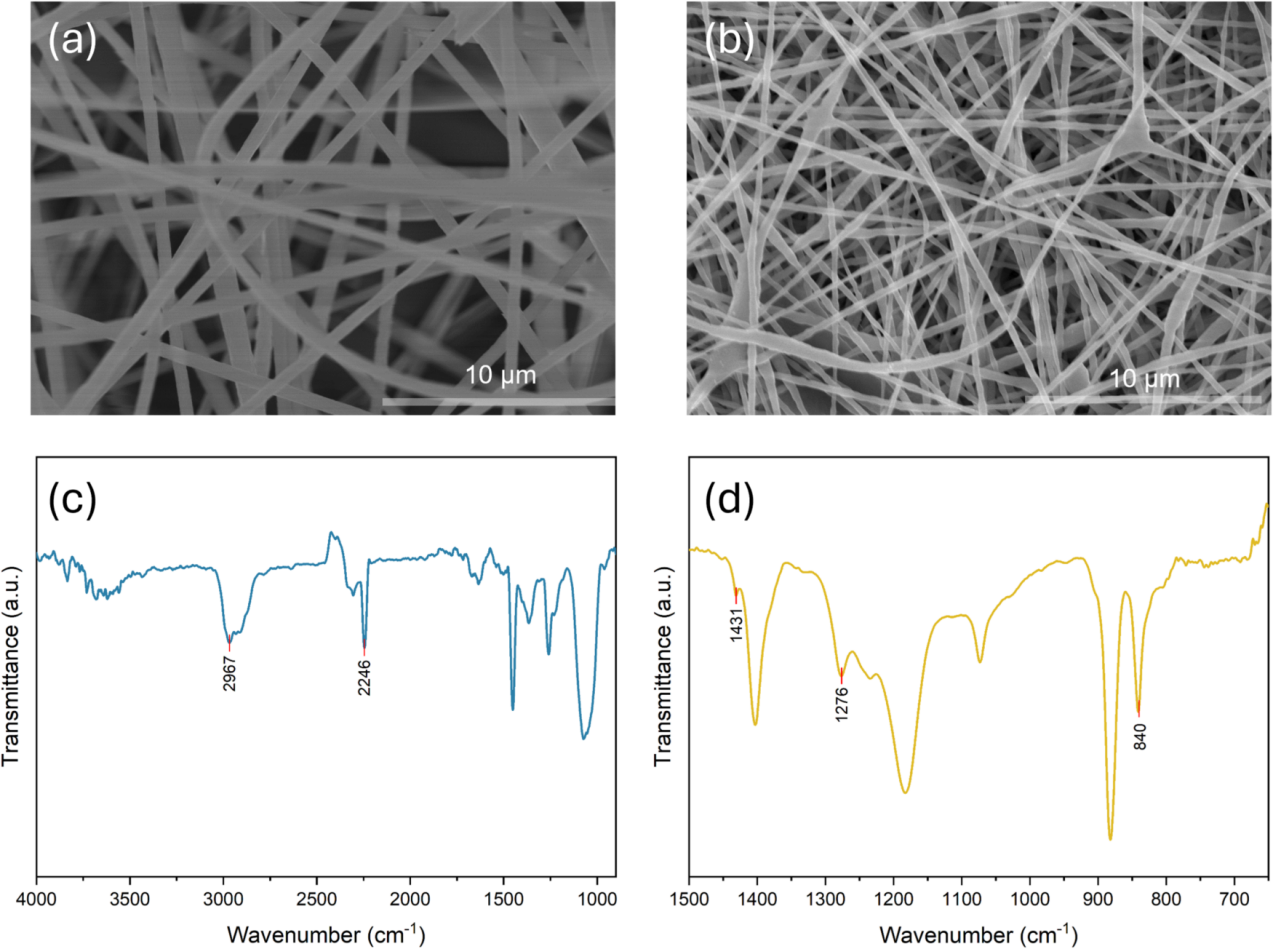
(a, b) SEM images and (c, d) FTIR spectra of (a, c) PAN and (b, d) PVDF.

The functional groups of PVDF and PAN were characterized using FTIR analysis (Figure 2c,d). For PVDF, owing to the kinetic preference, the α-phase is typically the most commonly formed crystalline structure during the melt or solution crystallization.^43^ However, the electrospinning process tends to promote the formation of the β-phase, which exhibits higher polarity, compared to the α-phase, due to the all-trans (TTTT) conformation and the preferential alignment of C–F dipoles. The β-phase formation is favored under the strong electric field and mechanical stretching involved in the electrospinning process, although the β-phase is less thermodynamically stable than the α-phase at normal conditions.^44^ Consistent with this expectation, the FTIR spectrum of the electrospun PVDF (Figure 2d) shows the diagnostic β-PVDF bands at 840, 1276, and 1431 cm^–1^, while no detectable signals at 614 and 763 cm^–1^, which are typical peaks of α-PVDF. This indicates the predominance of β-phase in the PVDF fibers.^45,46^ For PAN fibers (Figure 2c), the peaks at 2246 and 2967 cm^–1^ correspond to the stretching of nitriles and C–H bonds, respectively, revealing the successful formation of PAN nanofibers.

### 3.2 Electrochemical Characterizations for PAN and PVDF

Differences in chemical functionality between PAN and β-phase PVDF are expected to alter the Li^+^/anion interactions and the ion solvation structures and, consequently, the through-plane transport characteristics. We therefore determined the Li^+^ transference number (*t*_Li+_) in the Li||Li symmetric cells with PAN or PVDF ASEI-promoting membranes. A 10 mV DC bias was applied, and the current decay from the initial value to the steady-state value was recorded, following the Bruce-Vincent-Evans protocol; the impedance spectra were collected before and after the polarization to account for the interfacial resistance. As summarized in Figure 3a,b, the calculated t_Li+_ values are 0.64 for PAN and 0.58 for PVDF. Compared to the PAN fibers, the finer PVDF fibers form a denser crossover network with smaller pores, resulting in a larger electrochemically accessible area and, consequently, a higher areal density of C–F sites in contact with the electrolyte, although a single C–F termination is a weaker Lewis base than a nitrile group. By contrast, compared to the PVDF fibers, the PAN fibers, owing to their larger fiber diameter and looser junction density, present a smaller specific active area, although PAN can offer the stronger per-site Lewis basicity (–C≡N) than PVDF (−C–F sites).

**Figure 3:**
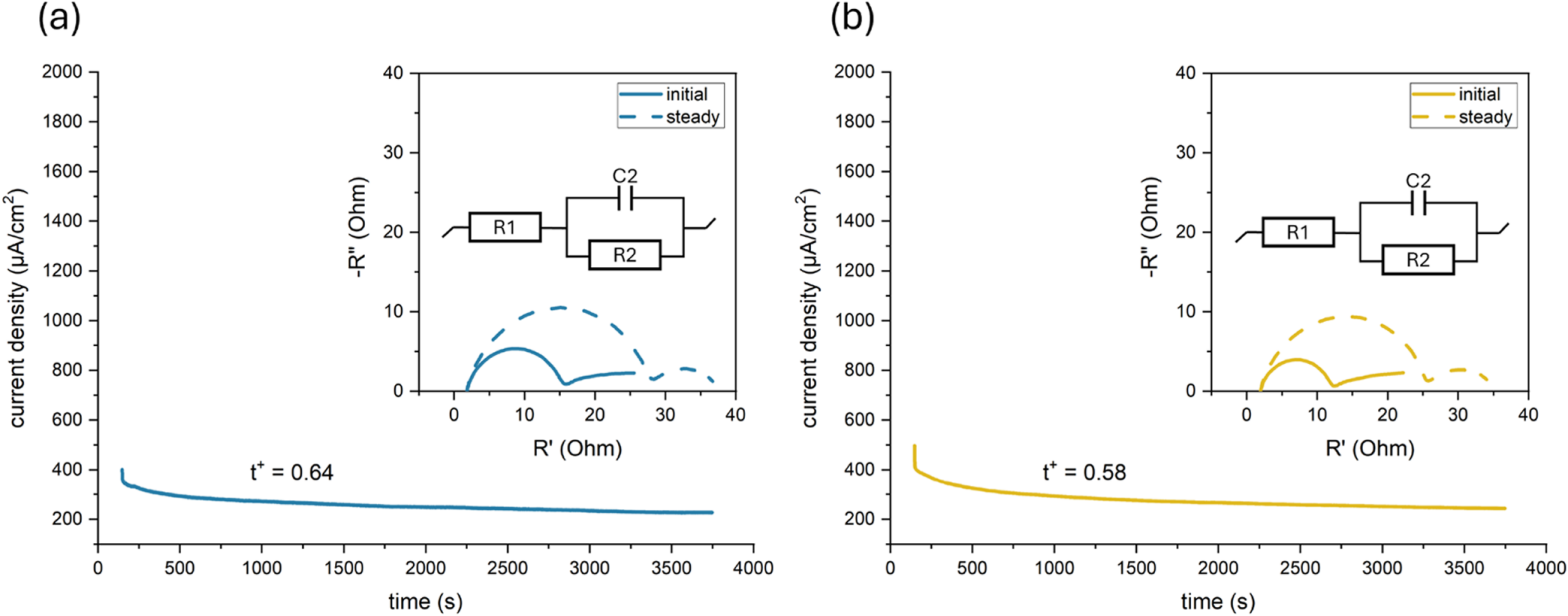
Lithium-ion transference number (*t*_Li+_) measurements of (a) PAN and (b) PVDF.

As *t*_Li+_ is a bulk through-plane metric, this difference in site population versus site strength leads to a higher *t*_Li+_ for PAN under a small potential-bias condition.^47,48^ In contrast, the EIS responses in Figure 3 isolate the interfacial kinetics at the contact between the electrospun fibers and Li electrodes. Here, the cell with the PVDF ASEI-promoting membrane displays a noticeably smaller charge-transfer resistance (*R*_ct_) compared to the cell with PAN. We attribute this behavior to the higher dielectric constant of β-PVDF (≈8)^49^ compared with PAN (2.82),^50^ which enhances the interfacial electronic polarization and, in turn, is expected to decrease the effective barrier of charge transfer.^51^

To further investigate both interfacial and bulk effects during cycling, galvanostatic charge–discharge (GCD) tests were conducted using a three-electrode configuration at a current density of 1 mA cm^–2^ with an areal capacity of 1 mAh cm^–2^. As shown in the voltage–time profiles (Figure S2), all samples exhibit an initial sharp voltage dip during the lithium plating process, corresponding to the nucleation overpotential, which is an indicator of the energy barrier associated with the formation of lithium nuclei on the current collector and thus predominantly sensitive to the interfacial properties under the high initial ion concentration. Consistent with the EIS trend, Cu@PVDF displays the lowest nucleation overpotential (0.113 V vs Li/Li^+^), followed by Cu@PAN (0.159 V), while bare copper (BC) shows the highest value (0.267 V), as summarized in Table S2.

It has been reported that the nucleation overpotential is inversely correlated to the size of lithium nuclei and that the initial nucleation morphology significantly impacts the subsequent lithium growth behavior.^52,53^ Guided by this framework, we examined the Cu substrates by SEM after galvanostatic Li plating for 50 s to probe the early stage deposition characteristics. As shown in Figure S3b, Cu@PAN exhibits sparse and isolated Li deposits with a substantial exposed Cu area, consistent with its relatively higher nucleation overpotential. In contrast, the Cu surface is largely covered with Li for Cu@PVDF (Figure S3c), indicating more extensive early stage deposition on the same time scale, in line with its lower nucleation overpotential.

To further evaluate how the initial deposition behavior evolves during the prolonged plating, Li-plated Cu electrodes were analyzed after galvanostatic deposition at 1 mA cm^–2^ for 22 h (Figure S4a,b). For Cu@PAN, the larger polarization can accelerate the parasitic electrolyte reduction and promote the growth of a thicker nascent SEI, which may hinder the lateral Li^+^ transport and favor the more localized growth during extended deposition.^54^ Such an evolution is often associated with nonuniform, dendrite-prone morphologies. In comparison, the lower overpotential and higher surface polarity of PVDF are expected to facilitate the interfacial ion transport and promote the more laterally uniform growth.^25,52^ Consequently, Li deposits on Cu@PVDF appear smooth and densely packed, providing a very uniform interface that is beneficial for improving the cycling stability of the ZELMBs.

### 3.3 Electrochemical Characterizations for the PAN/PVDF Composite Membrane

Building upon the above findings, a bilayer PAN/PVDF ASEI-promoting membrane was designed to enhance the Li^+^ transport within the electrolyte (inside the pores of the PAN/PVDF layer) and SEI layer, which can reduce the charge-transfer resistance at the electrode interface. To establish the fundamental structure–property basis, the porosity and electrolyte uptake were quantified (Tables S1 and Figure S1). The PAN/PVDF fibrous membrane exhibits porosity and electrolyte uptake values intermediate between those of PAN and PVDF fibers, suggesting that the bilayer architecture inherits complementary features from both polymers despite their distinct fiber morphologies.

Afterward, to determine the suitable layer configuration, Cu||Li half cells were fabricated using Cu@PAN + PVDF and Cu@PVDF + PAN and subjected to galvanostatic cycling at 1 mA cm^–2^ with an areal capacity of 1 mAh cm^–2^. As shown in Figure 4a, the copper substrates coated with only PAN or PVDF membranes maintain Coulombic efficiencies above 95% for approximately 130 cycles, both outperforming the bare copper substrate, which fails after around 110 cycles. However, their individual limitations, namely, the high nucleation overpotential of PAN and the low Li transference number of PVDF, still hinder the long-term performance. Remarkably, when PAN is positioned as the top layer and PVDF as the interfacial contact layer (i.e., Cu@PAN + PVDF), the cell exhibits a significantly extended lifespan, maintaining over 95% Coulombic efficiency for more than 160 cycles. In sharp contrast, the reverse configuration (Cu@PVDF + PAN) results in a premature failure with the CE dropping below 95% after only 100 cycles—even worse than using a bare Cu substrate. These contrasting results—despite the use of identical materials—highlight the profound impact of electrospun layer orientation on the electrochemical performance.

**Figure 4:**
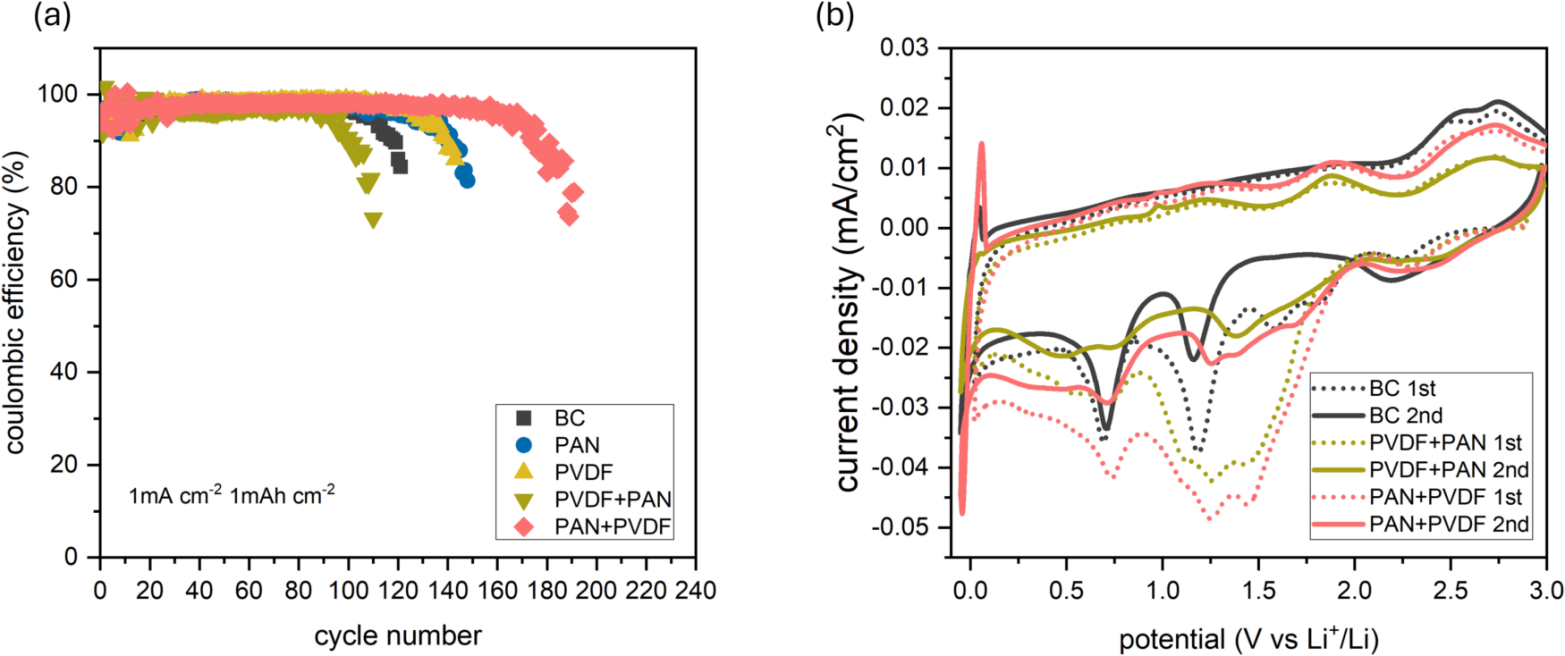
(a) Coulombic efficiency as a function of cycle number for Cu||Li coin cells. (b) CVs of BC, Cu@PVDF + PAN, and Cu@PAN + PVDF electrodes measured in a three-electrode system and scanned between 3.0 and 0.02 V in the first cycle (denoted as “1st”) and between 3.0 and −0.05 V in the second cycle (denoted as “2nd”) at a scan rate of 1 mV s^–1^.

Motivated by these results, the underlying mechanisms were examined through comparative electrochemical and morphological analyses of Li deposits on the Cu@PAN + PVDF and Cu@PVDF + PAN systems. First, we performed galvanostatic charge–discharge tests to compare the nucleation overpotentials of the two bilayer orientations. Figure S2 shows that Cu@PAN + PVDF exhibits a nucleation overpotential of 0.104 V, which is close to that of Cu@PVDF at 0.113 V. In contrast, Cu@PVDF + PAN shows a higher nucleation overpotential of 0.161 V, comparable to that of Cu@PAN at 0.159 V. The early stage top-view SEM images show the same trend. Cu@PAN + PVDF presents high-surface coverage of Li, similar to Cu@PVDF, while Cu@PVDF + PAN shows sparse and isolated Li deposits. These results indicate that the nucleation behavior of Li is mainly governed by the polymer membrane that is in direct contact with the Cu substrate.

Next, cyclic voltammetry (CV) was carried out at a scan rate of 1 mV s^–1^ within a voltage window between 3.0 and 0.02 V in the first cycle and between 3.0 and −0.05 V in the second cycle. The first cycle, limited to potentials above 0 V, was designed to evaluate the initial formation of the solid electrolyte interphase (SEI) on copper substrates. In the subsequent cycle, the voltage range was slightly extended to the potential region to further probe the reversibility of lithium plating-stripping on the Cu surface. As shown in Figure 4b, two major reduction peaks are observed during the negative scans. The peak between 1.2 and 1.7 V corresponds to the anion-derived reductions (e.g., TFSI^–^ and NO_3_^–^), which typically generates the inorganic species such as LiF and Li_3_N.^55^ These inorganic SEI components are mechanically robust and electronically insulating. Moreover, Li_3_N (and related nitride/oxynitride species) provides fast Li^+^ transport, while the interfacial LiF species is well-known to homogenize the interfacial fields and guide the uniform Li deposition.^56,57^

The second set of peaks, located around 0.5 to 0.9 V, arises from the reduction of ether-based solvents (DOL/DME in this study), forming the alkoxide species (ROLi) and poly­(ether)-type oligomers/polymers. Such organic components were reported to provide improved flexibility to accommodate large volume changes during lithium plating/stripping. However, their poor electrochemical stability can lead to SEI decomposition and continuous side reactions, resulting in the electrolyte consumption.^55,58^

A well-balanced inorganic–organic SEI is recognized as an ideal layer for enhancing both cycling stability and rate performance in LMBs.^13^ To deeply understand the individual roles of PAN and PVDF in SEI formation, we evaluated their electrochemical behaviors separately. As shown in Figure S6, Cu@PAN exhibits larger cathodic currents for both solvent and anion reduction compared to Cu@PVDF. This is consistent with the faster transport of solvated ion pairs through the more porous electrospun PAN fibers in comparison to the PVDF fibers. Notably, the inorganic/organic capacity ratio is markedly high with Cu@PAN, indicating a high anion-derived reduction and low solvent decomposition. This result stems from the strong –C≡N···Li^+^ binding at the PAN surface,^25^ which may enrich ion pairs at the surface, perturb their solvation shells, and facilitate the partial desolvation. This interpretation is supported by the absence of the distinct cathodic peak at ca. 0.75 V on Cu@PAN. In the second cycle, initial Li plating and stripping with low capacities were conducted to investigate the interfacial properties of the two electrospun ASEI-promoting membranes. Notably, the cathodic Li plating currents are comparable for both electrodes. However, the anodic Li stripping currents on Cu@PVDF are distinctly higher than those on Cu@PAN, indicating the more reversible Li plating-stripping responses on the former electrode (Cu@PVDF). This behavior is attributable to the higher dielectric constant and more uniformly distributed fiber junctions of β-PVDF compared to PAN, which mitigate the local field intensification and suppress the localized nucleation/growth, yielding a denser and flatter Li deposit—even in the presence of solvent-derived, organic-rich SEI components.

Interestingly, when PAN and PVDF are combined in a bilayer structure, their complementary properties yield a synergistic effect. In the Cu@PAN + PVDF configuration, the inorganic reduction peak (1.2–1.7 V) is significantly intensified compared with all other samples, indicating enhanced salt decomposition and inorganic SEI formation. This behavior is rationalized as follows: the PAN layer with stronger per-site Lewis basicity and larger pore size is expected to provide good ion accessibility and promote the Li^+^ transport with partial desolvation within the pores containing the electrolyte. Upon reaching the underlying PVDF, the high-dielectric environment lowers the bond-cleavage barrier within the coordinated anions, thereby accelerating the reduction of the anions. In contrast, the Cu@PVDF + PAN structure suppresses the reduction currents. The top PVDF layer with a high-dielectric constant and small pores cannot provide the partially desolvated ions before reaching the metal/SEI interface. The resulting CV profile is effectively PAN-like, with an additional cathodic feature near about 0.75 V, which is attributable to the thin PAN layer. In summary, PAN is believed to contribute regardless of the position: its porous, amorphous network and –C≡N···Li^+^ interactions enable ion access and facilitate the partial desolvation of ions. PVDF is effective only when it contacts the Cu interface; its high-dielectric characteristic can enhance the interfacial charge transfer and promote the anion bond cleavage, yielding an inorganic, LiF-rich SEI. Thus, layer order matters; Cu@PAN + PVDF can leverage both functions, whereas Cu@PVDF + PAN largely forfeits the benefit of PVDF.

Nonetheless, the Cu substrates with both composite membranes outperform the bare Cu by producing a more inorganic-rich SEI and exhibiting more complete SEI formation, as evidenced by the significantly lower reduction currents in the second cycle. Without a protective ASEI-promoting membrane, the Li^+^ flux at the bare Cu interface remains unregulated, producing a nonuniform SEI prone to cracking and repeated solvent decomposition. Notably, the current density near 0 V for Cu@PAN + PVDF is significantly higher than those for the other two samples, indicating that this electrode facilitates stable electrode–electrolyte interfacial kinetics and enables lithium nucleation at lower overpotentials. Collectively, these results demonstrate that the bilayer structure not only promotes the formation of an inorganic-rich SEI but also stabilizes the interfacial reactions, ultimately leading to uniform and reversible lithium deposition/stripping.

To further validate the results obtained from CV, X-ray photoelectron spectroscopy (XPS) is performed to analyze the chemical composition of the SEI layers formed on Cu electrodes. All samples were subjected to voltage scans within the range of 3.0–0.02 V, consistent with the CV setup. Here, special attention was given to two key inorganic SEI components, lithium fluoride (LiF) and lithium nitride (Li_3_N), for their superior properties in facilitating the uniform Li plating in LMBs.^59–61^

From Figure S8a,b, all samples exhibit a higher portion of Li_*x*_CF_*y*_ species at the SEI surface, while the F 1s spectra at a depth of 60 nm are dominated by LiF. The situation can be rationalized by the following mechanism. In the early stages of SEI formation, low interfacial impedance facilitated the multielectron reduction of TFSI^–^ to produce LiF on the current collector surface.^62^ However, as the SEI grew thicker, the transport of ion pairs became hindered, limiting further decomposition of salts. Subsequently, solvent molecules were decomposed and reacted with the reduction products of TFSI^–^, thereby forming the organic-rich films.^55^ Consequently, a LiF-rich inner layer and a C–F-rich outer layer were established, consistent with the F 1s XPS depth profiles shown in Figure S7a–c. Among all samples, Cu@PAN + PVDF exhibited the highest amounts of LiF, followed by Cu@PVDF + PAN, whereas the bare copper showed the lowest content of inorganic SEI components. This result aligns very well with the CV results, in which Cu@PAN + PVDF displayed the most pronounced TFSI^–^ reduction peak.

In contrast, NO_3_^–^ was initially decomposed to form LiNO_2_ and then was further reduced into Li_3_N and Li_2_O.^63^ From Figure S7d–f, the Cu@PAN + PVDF electrode demonstrates the strongest intensity of Li_3_N in both surface and subsurface regions, following the same trend as LiF. Notably, the Cu@PAN + PVDF electrode exhibits the highest Li_3_N/LiN_*x*_O_*y*_ ratio (Figure S8c,d), suggesting that this bilayer configuration can provide a favorable interface for the complete reduction of nitrate species. Collectively, these findings further support the role of the electrospun PAN + PVDF bilayer membrane in promoting a stable, inorganic-rich SEI.

More importantly, with the synergistic contributions from PAN and PVDF, Cu@PAN + PVDF||Li exhibits the highest exchange current density (0.426 mA cm^–2^) among all of the cells (Figure S9 and Table S4). This suggests its highly efficient charge transfer and reduced interfacial overpotential, which are favorable for the uniform lithium deposition.^64,65^ In contrast, both BC and Cu@PVDF + PAN show relatively low exchange current densities of 0.141 and 0.077 mA cm^–2^, respectively. The result reflects their poor electrode–electrolyte interfacial properties. This limited the interfacial kinetics, likely due to the inadequate SEI composition and the absence of high-dielectric electronic polarization at the interface from the β-PVDF layer to promote the charge transfer of Li plating/stripping.

To gain deep insight into the influence of the polymer layer configuration, we further examined the lithium deposition behavior. Cross-sectional SEM analysis was conducted on Li-plated Cu@PAN + PVDF and Cu@PVDF + PAN, as presented in Figure 5a–d. First, electrodes were plated with Li at a constant current density of 1 mA cm^–2^ for 22 h in Cu||Li cells (Figure 5a,b). Notably, both electrospun layers were mechanically widened when the plating was progressed, indicating that the interlayers actively participated throughout the whole deposition process rather than only during the onset of the deposition period.

**Figure 5:**
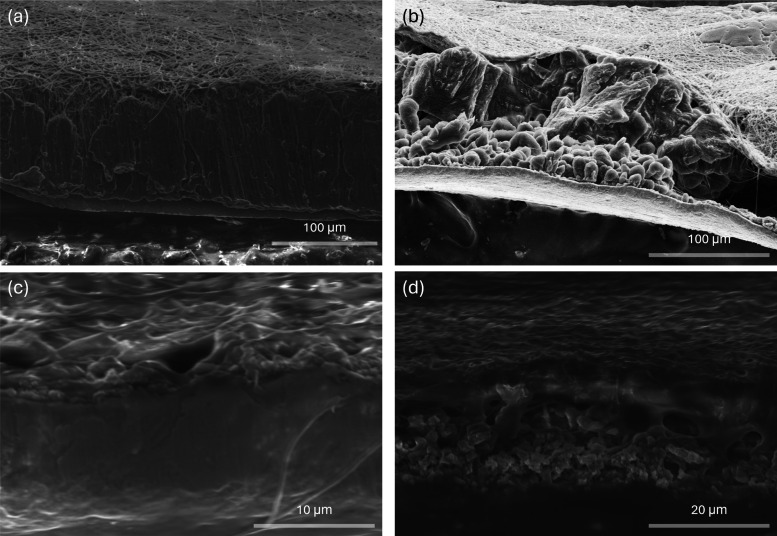
(a, b) Cross-sectional SEM images of the Li deposits via plating at 1 mA cm^–2^ for 22 h; (c, d) after a plating/stripping protocol: plating at 1 mA cm^–2^ to 1 mAh cm^–2^, then stripping at 1 mA cm^–2^ to 0.5 mAh cm^–2^ in Cu||Li cells. Results are shown for (a, c) Cu@PAN + PVDF and (b, d) Cu@PVDF + PAN.

The Cu@PAN + PVDF||Li cell—where PVDF served as the interfacial contact layer, exhibited a dense and uniform lithium deposit morphology, resembling that formed in the Cu@PVDF||Li system (Figure S4a). This suggests that the PAN + PVDF configuration successfully inherited the functionalities of PVDF. Moreover, the presence of an inorganic-rich SEI (LiF and Li_3_N) further contributed to the regulation of the Li^+^ flux and suppressed the dendritic growth, resulting in the compact and layered lithium deposit. The theoretical thickness of an ideal, uniform, and dense metallic Li deposited at 1 mA cm^–2^ to an areal capacity of 22 mAh cm^–2^ is 107 μm if the density of metallic Li (ρ_Li_) is 0.534 g cm^–3^. Thus, the ideal total thickness of membrane +Li deposit is about 117 μm since the thickness of the protective fibrous membrane is ∼10 μm. As summarized in Table S3, the Cu@PAN + PVDF electrode exhibited the thinnest lithium deposit, with a measured thickness of about 120 μm, which is very close to the ideal value. This strong consistency suggests that the PAN + PVDF bilayer membrane effectively suppressed the excessive volume expansion during cycling and enabled the precise control of lithium deposit thickness corresponding to a given areal capacity. In contrast, the Cu@PVDF + PAN||Li cell in Figure 5b displays a distinctly different morphology characterized by a vertically segregated structure. At the bottom (near the Cu interface), spherical lithium nuclei are observed—likely due to the high nucleation overpotential induced by PAN as the interfacial layer, as previously discussed in Section 3.2 (Figure S4b). Above this layer, large lithium grains are present, forming a loosely packed, uneven structure. As a result, the overall lithium morphology resembles a “lollipop-like” structure, initiating from the point nuclei and proceeding with unconstrained lateral and vertical growth. This type of deposition not only introduced significant volume fluctuations (with a deposit thickness of 172 μm) but also impaired the reversibility of lithium stripping due to the poor structural integrity and inconsistent interfacial contact.

This hypothesis is further supported by the half-stripping experiment. Here, Cu@PAN + PVDF||Li and Cu@PVDF + PAN||Li cells were first plated with Li at a current density of 1 mA cm^–2^ to an areal capacity of 1 mAh cm^–2^, followed by a partial stripping process at the same current density to a capacity of 0.5 mAh cm^–2^. The cross-sectional SEM images of the Li-plated Cu electrodes after the half-stripping process are shown in Figure 5c,d. For the Cu@PAN+PVDF configuration (Figure 5c), the residual Li deposits exhibit a uniform morphology without pronounced void formation or structural separation, indicating a gradual and spatially homogeneous removal of Li. Such a behavior is believed to promote a complete and reversible lithium utilization, effectively suppressing the dead Li formation and enhancing the long-term cycling stability of the cell. In sharp contrast, on the Cu@PVDF + PAN electrode (Figure 5d), the lithium deposit presents a compact upper region over a loosely packed, granular sublayer; the pronounced midlayer voids further indicate the nonuniform stripping of Li on this electrode.

A schematic explanation of this observation is proposed in Figure 6. During charging, Li ions penetrated the bilayer membrane and were reduced to form Li nuclei at the Cu surface contacting the fibers. Note that the bilayer membranes were found to promote the formation of an inorganic-rich SEI and stabilize the interfacial reactions, especially for the PAN + PVDF configuration (i.e., Cu@PAN + PVDF). For the nucleation and growth of metallic Li in the configuration of PAN + PVDF, the nitrile groups in the top PAN layer are expected to facilitate the Li^+^ transport and partial desolvation; meanwhile, the C–F bonds in the PVDF bottom layer are used to regulate the partially desolvated Li^+^ flux to get a uniform and dense Li deposit. As a result, the plating/stripping reversibility of metallic Li is synergistically promoted by this PAN + PVDF bilayer membrane.

**Figure 6:**
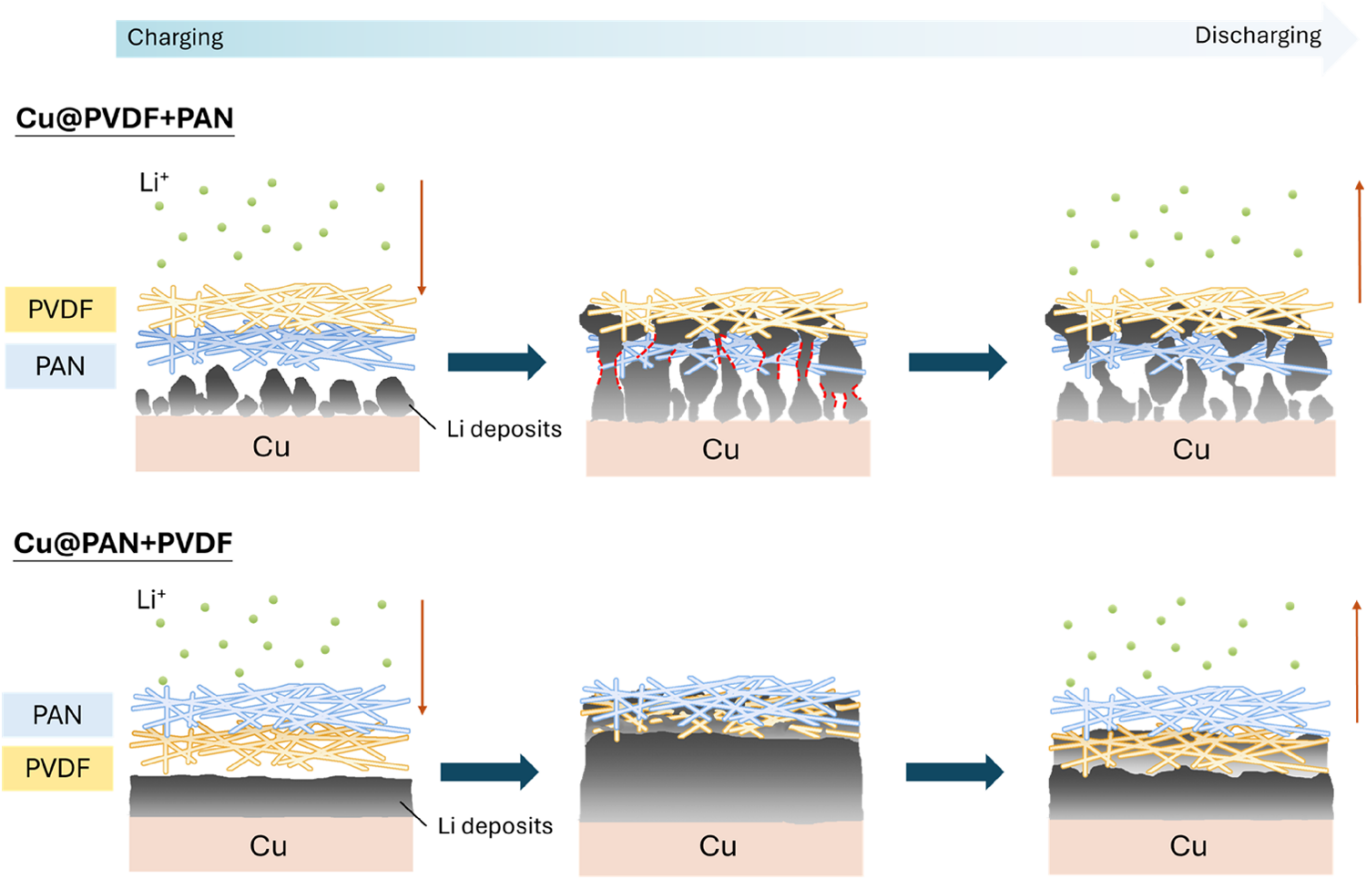
Schematic illustration of the lithium plating/stripping mechanism on Cu@PVDF + PAN and Cu@PAN + PVDF, highlighting the distinct nucleation, growth, and stripping behaviors.

In contrast, when the configuration of PVDF + PAN is used (Cu@PVDF + PAN), the relatively dense PVDF upper layer increases the Li^+^ transport resistance but cannot offer a partial desolvation function. In addition, the underlying, highly porous PAN layer favors the nucleation of isolated Li, leading to the growth of a relatively porous/loosely packed Li deposit. As a result, during the stripping process, the current density concentrates in the relatively porous/loosely packed region (highlighted by the red dashed line), accelerating the lithium removal and leading to delamination between the top and bottom lithium grains. Consequently, the remaining isolated lithium at the top becomes electrochemically inactive, which is commonly referred to as “dead Li.” This contributes to the irreversible capacity loss and largely shortens the cycle life in ZELMBs.

### 3.4 Full-Cell Performance of PAN + PVDF-Modified ZELMBs

To evaluate the practical applicability of ZELMBs using the PAN+PVDF ASEI-promoting membrane, full cells were assembled using LiFePO_4_ (LFP) as the positive electrode material. The cells were charged/discharged at 0.1C for the first cycle and subsequently at 0.5C/1C for the following cycles. As shown in Figure S10a, the two cells deliver a similar initial capacity (138.95 mAh g^–1^ for Cu@PAN + PVDF||LFP; 138.02 mAh g^–1^ for the Cu||LFP), and a larger capacity loss during the first three cycles was observed for Cu@PAN + PVDF||LFP compared to Cu||LFP. This can be attributed to the formation of an inorganic-rich SEI induced by the PAN + PVDF membrane, which leads to high lithium consumption initially. Thereafter, the stabilized interface suppresses the further capacity decay, so Cu@PAN + PVDF||LFP maintains a stable Coulombic efficiency (CE, ∼100%), achieving a capacity retention of 51.6% over 70 cycles. In contrast, the Cu||LFP cell retains only 42.2% of its initial capacity over the same period, with CE falling below 95% after 100 cycles. The charge/discharge voltage profiles (Figure S10b,c) further reveal that the Cu@PAN + PVDF||LFP cell exhibited a smaller voltage hysteresis, indicating better interfacial stability and a lower IR drop, compared to the Cu||LFP.

Nevertheless, the cycle life of ZELMBs remains limited with the loss of active lithium being one of the primary contribution factors. To mitigate this issue but maintain the high energy density characteristics, a prelithiation strategy was employed.^66^ Ultrathin lithium layers were electrochemically deposited onto a bare Cu foil (Li/Cu) and a Cu@PAN + PVDF electrode (Li/Cu@PAN + PVDF). The current density was set at 1 mA cm^–2^ for 2 h, corresponding to an N/P ratio of 1.3. After that, the thin metallic lithium LMBs were assembled with the prelithiated negative electrodes and the LFP positive electrodes, denoted as “Li/Cu||LFP” and “Li/Cu@PAN + PVDF||LFP”. As shown in Figure 7, the prelithiation process significantly extended the cycling life for both cells by compensating for the Li^+^ consumption during the SEI evolution. Remarkably, Li/Cu@PAN + PVDF||LFP demonstrates superior long-term stability, with a capacity retention of 57.68% after 200 cycles, versus 27.7% for Li/Cu||LFP. This improvement arises from the reduced charge-transfer barrier and the uniform Li^+^ flux enabled by the electrospun PAN + PVDF ASEI-promoting membrane, which enhances the homogeneous plating/stripping and prolongs the cycle life relative to a bare Cu substrate.

**Figure 7:**
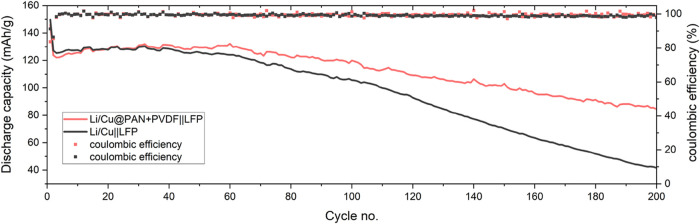
Cycling performance of Li/Cu||LFP and Li/Cu@PAN + PVDF||LFP cells made from the prelithiated substrates prepared at 1 mA cm^–2^ for 2 h (N/P ratio = 1.3). All cells were tested at 0.5*C*/1C, with the first cycle conducted at 0.1C.

## 4 Conclusions

This study demonstrated the combination of electrospun PAN and PVDF as an ASEI-promoting membrane for the negative electrode of ZELMBs and revealed that the sequence of the dual-layer structure plays a decisive role in cell performance. PAN fibers with strong Lewis-base sites are believed to promote the partial desolvation of ions, resulting in the formation of an inorganic-rich SEI. In contrast, the dense morphology and weak basicity of PVDF fibers lead to a low Li^+^ transference number and an organic-rich SEI. When PVDF is placed as the electrode contact layer, the dielectric properties of β-PVDF effectively reduce the charge-transfer resistance and lower the nucleation barrier of Li plating. As a result, the Cu@PAN + PVDF shows the LiF- and Li_3_N-rich SEI, as well as uniform lithium deposits. Comparatively, the opposite Cu@PVDF + PAN electrode forfeits the advantage of dielectric PVDF, yielding the spherical nuclei similar to those observed on the Cu substrate coated with the single PAN layer. Moreover, the half-stripping experiment proposed in this work offers a novel approach to examine the structural integrity and reversibility of metallic lithium deposits. SEM analysis after the partial delithiation revealed that the Cu@PAN + PVDF maintained a flat, compact lithium layer, while the Cu@PVDF + PAN developed the loosely packed morphology and distinct midlayer voids, signifying the localized stripping and the formation of inactive “dead Li.” This diagnostic approach not only elucidates the evolution of Li morphology during cycling but also provides a practical methodology for evaluating the interfacial stability in the future ZELMB research.

Overall, this study deepens the understanding of the artificial SEI structure–function relationships and presents a facile design strategy for suppressing the dendrite growth, promoting the homogeneous Li deposition, and extending the cycle life of ZELMBs. While the present work focuses on isolating dual-layer orientation effects under excess-electrolyte conditions, extending the evaluation to lean-electrolyte operation will be an important next step toward the practical implementation.

## Supplementary Material



## Notes

Additional experimental details, materials, and methods, including photographs of the experimental setup (PDF)
