# Supplementary material for: Electrospun Polyacrylonitrile/Polyvinylidene Difluoride Bilayer Promoting the Uniform Lithium Deposition/Stripping in the Zero-Excess Lithium Metal Batteries
Source: ACS Appl Mater Interfaces. 2026 Mar 27;18(13):19047–58. doi: 10.1021/acsami.5c25604 (PMC13067238; doi:10.1021/acsami.5c25604)
Supplement: Supplementary file 1 [file am5c25604_si_001.pdf]

Supporting Information

Electrospun polyacrylonitrile/polyvinylidene difluoride bilayer promoting the uniform lithium deposition/stripping in the zero-excess lithium metal batteries

Yu-Jun Wei[a], Ai-Ling Huang[a], Hao-Yu Ku[a], Hsiang-Sheng Wei[a], Chi-Yu Lai[a], Chi-Chang Hu[a, b, c, *]

[a] *Department of Chemical Engineering, National Tsing Hua University, Hsin-Chu 300044, Taiwan*

[b] *College of Semiconductor Research, National Tsing Hua University, Hsin-Chu 300044, Taiwan*

[c] *College of Sustainability, National Tsing Hua University, Hsinchu 300044, Taiwan*

*Corresponding Author: Chi-Chang Hu, NTHU Chair Professor
Department of Chemical Engineering
National Tsing Hua University
101, Section 2, Kuang-Fu Road
Hsin-Chu, 300044, Taiwan
Email: cchu@che.nthu.edu.tw

This supporting information includes 10 figures and 4 tables.

Table S1. The preparation parameters of all electrospun samples.

Sample	Flow rate (mL h^{-1})	Distance (cm)	Voltage (V)	Temperature (^{o}C)	Volume (mL)	Thickness (μm)	Areal mass (mg cm^{-2})
PAN	0.5	16.5	12500	38	1.2	10.10	0.195
PVDF	0.9	13	12500	38	2	10.37	0.376
PAN/PVDF	0.5/0.9	16.5/13	12500	38	0.6/1	10.17 (5.085/5.085)	0.286

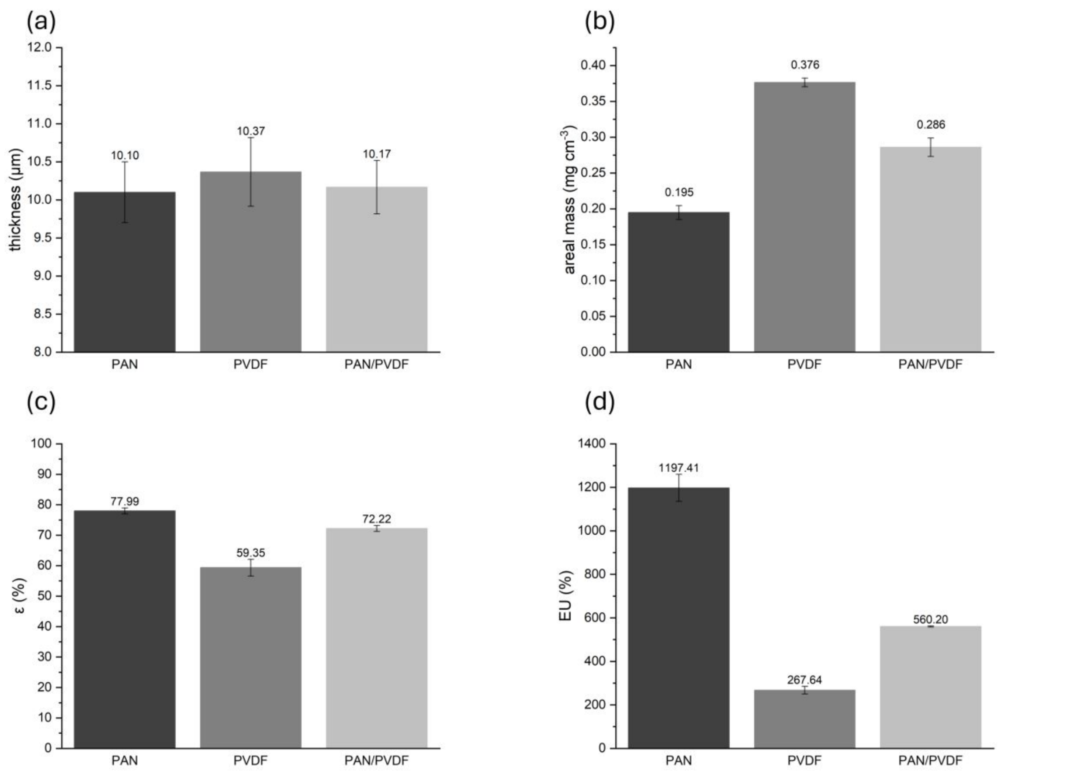

Figure S1. (a) Thickness, (b) areal mass, (c) porosity (ε %), and (d) electrolyte uptake (EU %) of all electrospun membranes.

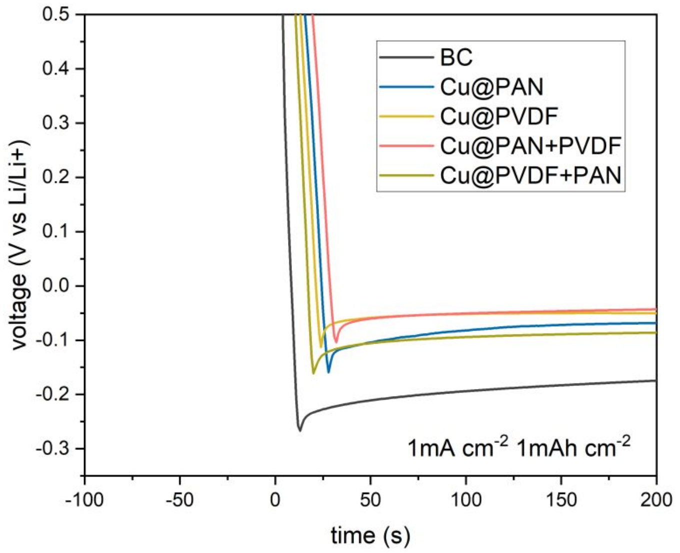

Figure S2. The nucleation overpotentials of the first lithiation cycle on various substrates at a constant current density of 1 mA cm^{-2} and a capacity of 1 mAh cm^{-2}.

Table S2. The nucleation overpotentials of Li deposition at 1 mA cm^{-2} on various substrates investigated in this work (the bare Cu is denoted as BC).

Sample	BC	Cu@PAN	Cu@PVDF	Cu@PVDF+PAN	Cu@PAN+PVDF
Nucleation Overpotential (V vs. Li/Li$^+$)	0.267	0.159	0.113	0.161	0.104

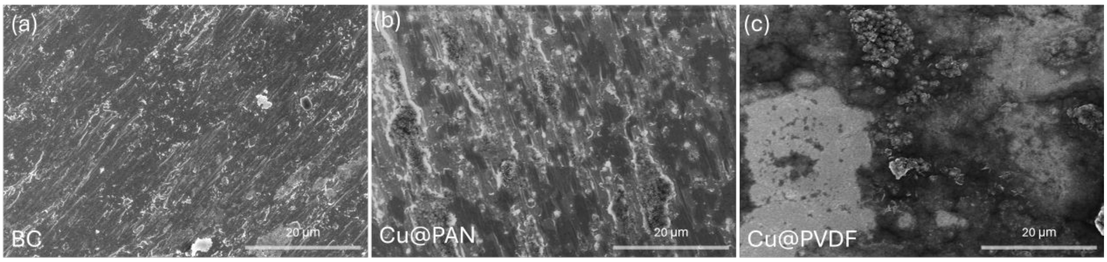

Figure S3. The top-view SEM images of (a) a bare copper foil and (b, c) the early-stage Li nucleation morphologies of (b) Cu@PAN and (c) Cu@PVDF. The latter two electrodes were plated at a constant current density of 1 mA cm^{-2} for 50 s.

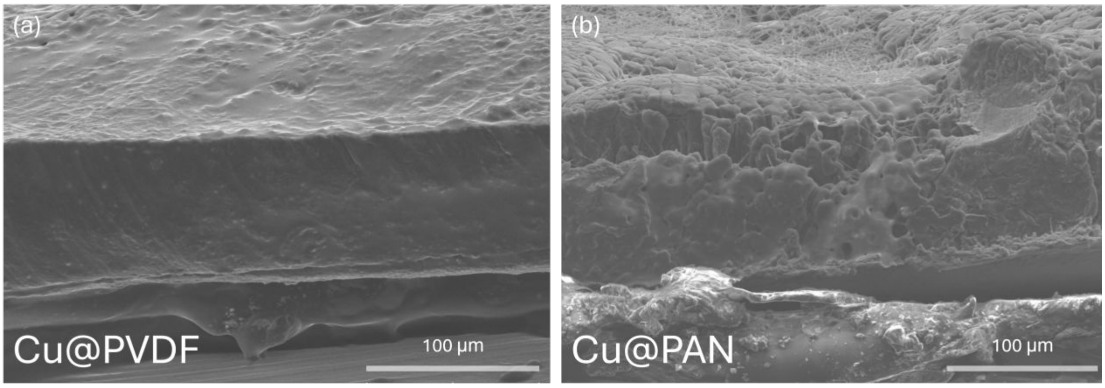

Figure S4. The cross-sectional SEM images of Li deposits on (a) Cu@PVDF and (b) Cu@PAN. Both deposits were plated at a constant current density of 1 mA cm^{-2} for 22 h.

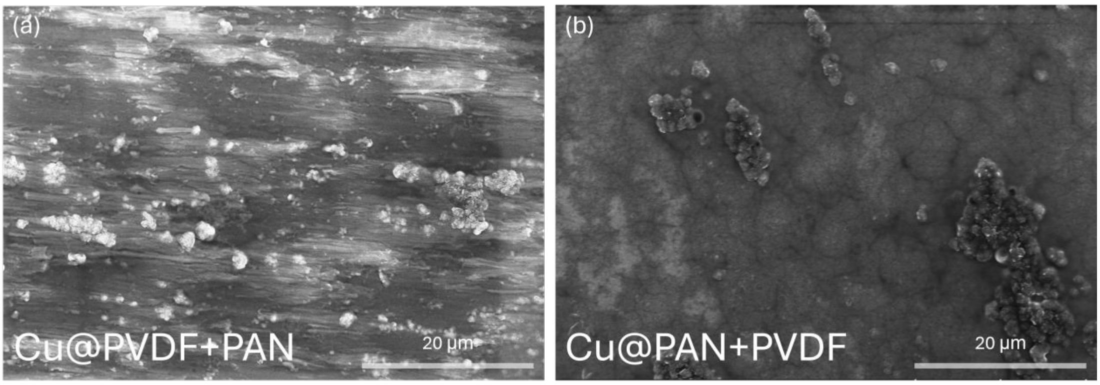

Figure S5. The top-view SEM images of (a) Cu@PVDF+PAN and (b) Cu@PAN+PVDF. Both electrodes were plated at a constant current density of 1 mA cm^{-2} for 50 s.

Table S3. The thickness of lithium deposits on copper substrates with various ASEI-promoting membranes for the electroplating at a constant current density of 1 mA cm^{-2} for 22 h. Notably, the thickness was determined using the copper foil (10 μm) as an internal reference scale to minimize underestimation caused by the limited depth of field in SEM imaging.

Sample	Cu@PAN	Cu@PVDF	Cu@PVDF+PAN	Cu@PAN+PVDF
Thickness (μm)	172	151	191	120

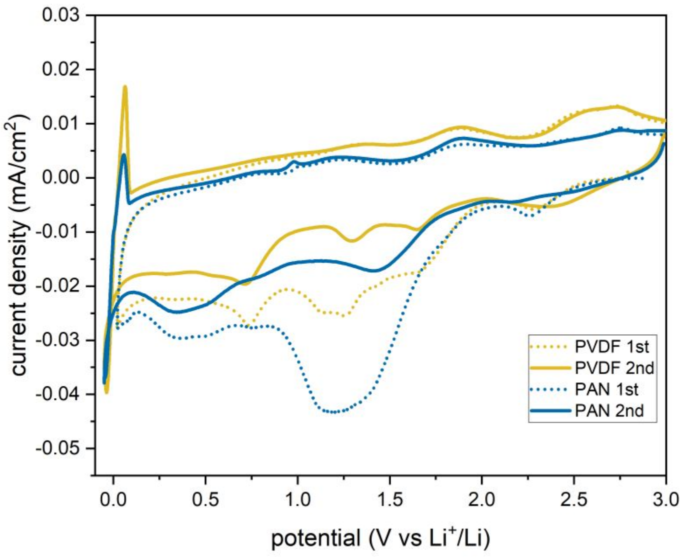

Figure S6. CVs on Cu@PVDF and Cu@PAN between 3.0 V and 0.02 V in the first cycle (labeled as “1st") and between 3.0 V and -0.05 V in the second cycle (labeled as "2nd").

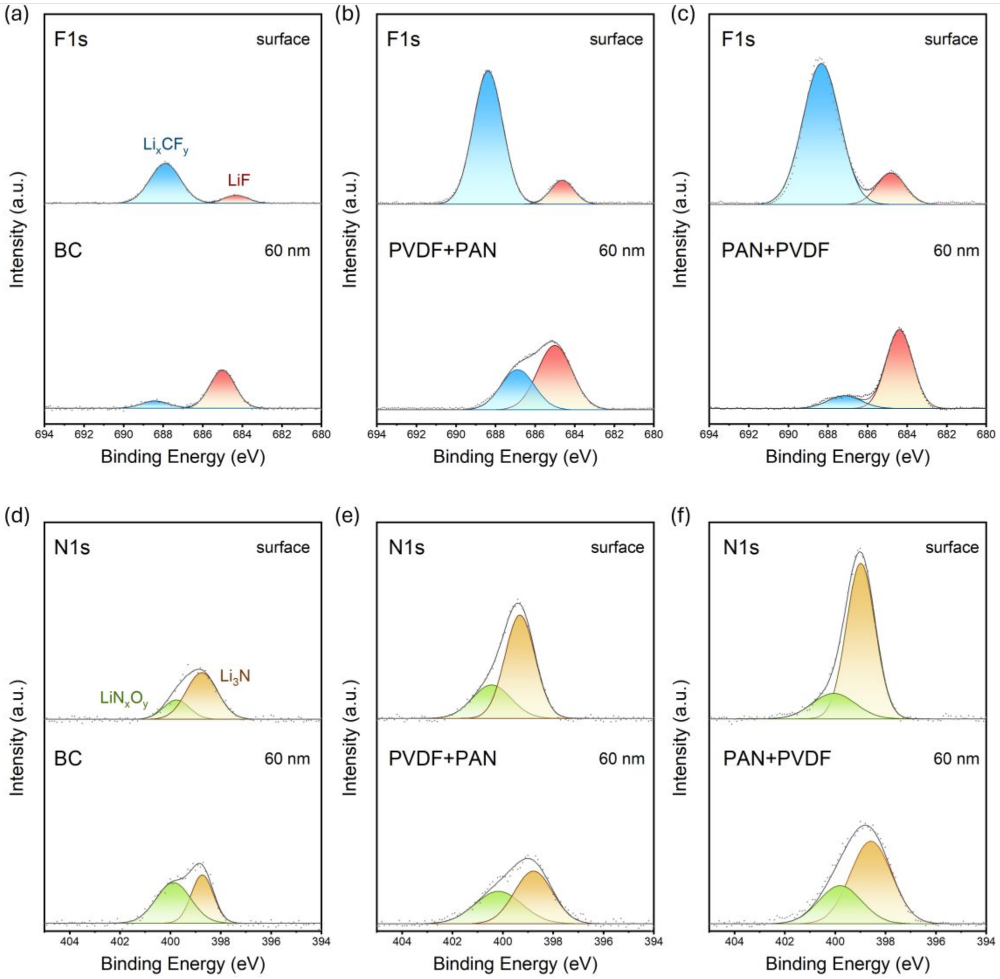

Figure S7. (a-c) F 1s XPS spectra and (d-f) N 1s XPS spectra of the SEIs formed on BC, Cu@PVDF+PAN, and Cu@PAN+PVDF after 1 CV cycling between 3.0 and 0.02 V; the XPS spectra were obtained at the electrode surface and at a depth of 60 nm.

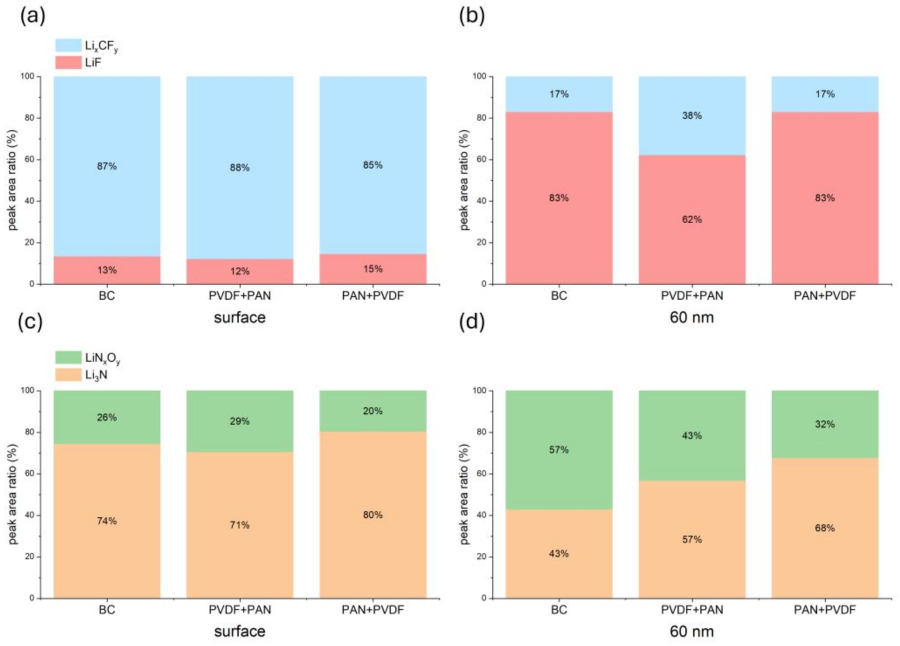

Figure S8. Fitted peak area ratios of (a, b) F1s and (c, d) N1s at (a, c) surface (0 nm) and (b, d) at the depth of 60 nm.

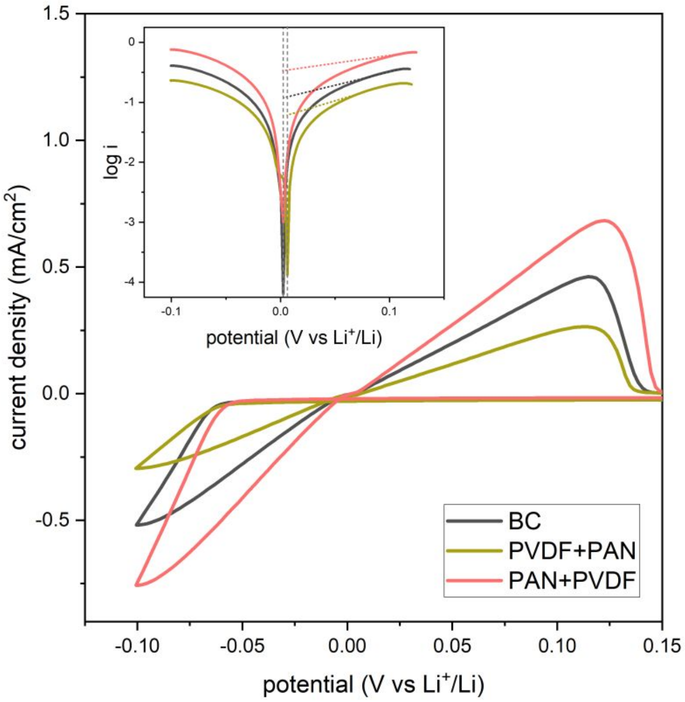

Figure S9. CVs between 3.0 V and -0.1 V and log(i)-E plots measured at a scan rate of 1 mV s^{-1} for the Cu||Li cells under a three-electrode configuration.

Table S4. Exchange current density (j_0) estimated from the Tafel plot.

Sample	BC	Cu@PVDF+PAN	Cu@PAN+PVDF
Exchange current density (mA cm^{-2})	0.141	0.077	0.426

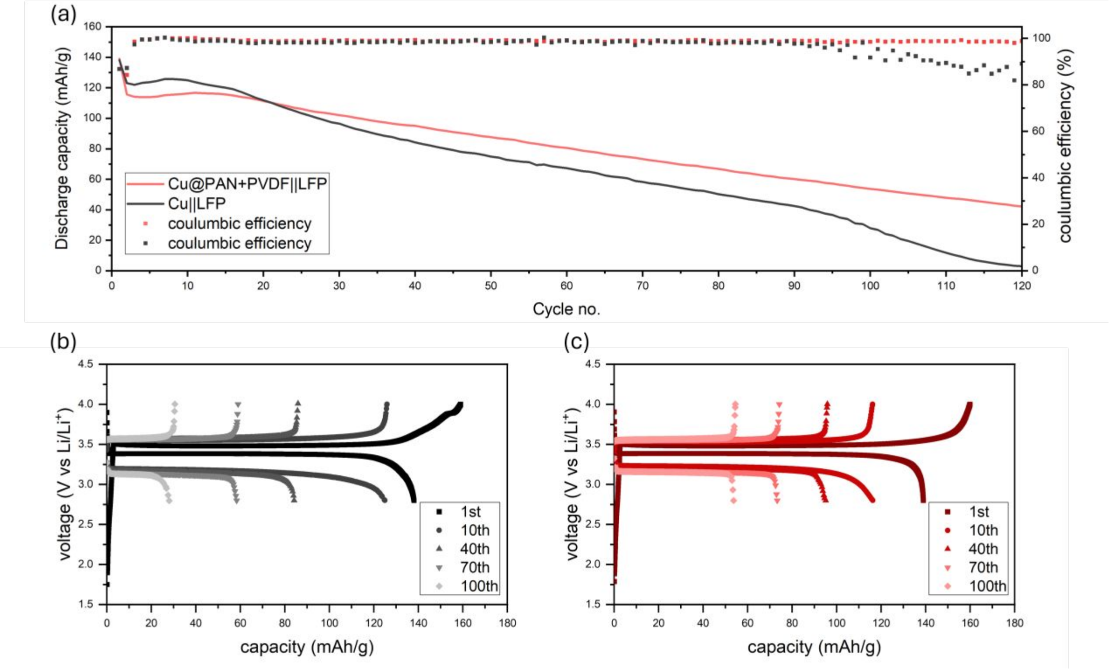

Figure S10. (a) Cycling performance of Cu@PAN+PVDF||LFP and Cu||LFP, and the GCD cell voltage profiles of (b) Cu||LFP and (c) Cu@PAN+PVDF||LFP ZELMBs at the charge/discharge rates of 0.5C/1C. The first cycle was conducted at 0.1C/0.1C.
